# Symmetry issues in the hybridization of multi-mode waves with resonators: an example with Lamb waves metamaterial

**DOI:** 10.1038/srep13714

**Published:** 2015-09-03

**Authors:** Matthieu Rupin, Philippe Roux, Geoffroy Lerosey, Fabrice Lemoult

**Affiliations:** 1 Institut Langevin, ESPCI ParisTech and CNRS UMR 7587, PSL Research University, 1 rue Jussieu, 75005, Paris, France; 2 Institut des Sciences de la Terre, UMR 5275, Université Joseph Fourier, Grenoble, 38000, France

## Abstract

Locally resonant metamaterials derive their effective properties from hybridization between their resonant unit cells and the incoming wave. This phenomenon is well understood in the case of plane waves that propagate in media where the unit cell respects the symmetry of the incident field. However, in many systems, several modes with orthogonal symmetries can coexist at a given frequency, while the resonant unit cells themselves can have asymmetric scattering cross-sections. In this paper we are interested in the influence of symmetry breaking on the hybridization of a wave field that includes multiple propagative modes. The *A*_0_ and *S*_0_ Lamb waves that propagate in a thin plate are good candidates for this study, as they are either anti-symmetric or symmetric. First we designed an experimental setup with an asymmetric metamaterial made of long rods glued to one side of a metallic plate. We show that the flexural resonances of the rods induce a break of the orthogonality between the *A*_0_/*S*_0_ modes of the free-plate. Finally, based on numerical simulations we show that the orthogonality is preserved in the case of a symmetric metamaterial leading to the presence of two independent polariton curves in the dispersion relation.

Metamaterials refer to man-made composite media that are composed of an ensemble of sub-wavelength unit cells. When modifying the characteristics of the unit cell, it is possible to impart effective propagation properties to the medium that are not available in natural materials^1^,^2^. To this end, the use of resonant unit cells is of particular interest for the wide range of degrees of freedom it offers for the design of so-called ‘locally resonant’ metamaterials^3^,^4^. Similar to the case where a photon interacts with an atom, the physics that governs this kind of locally resonant metamaterial stems from the interactions between the scattered and unscattered parts of an incident wave that creates Fano interferences^5^ around the resonance frequency of the unit cell. By tuning the unit-cell resonance, it is possible to obtain media with effective parameters (e.g., permeability and/or permitivity for electromagnetic waves, or mass and/or modulus for acoustic waves) that can be negative^4^,^6^,^7^,^8^, high^9^,^10^,^11^,^12^ or null^13^. Locally resonant metamaterials have also been used to design media with both of these effective parameters being negative; this leads to the class of negative index media^14^,^15^,^16^,^17^, which is the basis of the concept of the perfect lens^18^,^19^. For a scalar wavefield, a locally resonant metamaterial has a dispersion that is induced by a local resonance that shows two hybrid modes when observed in the frequency-wavenumber space. This results from the anti-crossing effects between a continuum of frequency with a localized frequency, which are also known as polariton branches^20^. An interesting question then arises: what about a medium with multiple propagative modes that interacts with multiple localized resonances?

To answer this question, we are interested in the study of elastic guided waves that propagate in a thin aluminum plate (i.e., the host medium) that is coupled with a group of long aluminum rods (i.e., the local resonators), as shown in Fig. 1a. For the host medium, an elastic plate is a good example of a medium that has multiple propagative modes, and was already used in the past in the context of metamaterials or phononic crystals^21^,^22^,^23^,^24^,^25^,^26^. Indeed, a thin plate refers to a plate with a small thickness *h* compared to the wavelength *λ* in the bulk elastic medium. In the low-frequency regime, three propagative guided modes coexist: the *A*_0_ Lamb mode that corresponds to a flexural motion of the plate (i.e., quasi-transverse displacement), the *S*_0_ Lamb mode that mostly corresponds to an in-plane compressional motion of the plate (i.e., quasi-longitudinal displacement) and the *SH*_0_ mode that corresponds to an in-plane horizontal shear mode. These modes result from the boundary condition problem applied to the bulk elastic waves (i.e., both transverse and longitudinal) and set the orthogonal basis of the guided wave solutions in the plate^27^.

For the local resonators, finite length aluminum rods are also good candidates. Indeed, an infinitely long aluminum rod has similar propagative modes as a thin plate except for the horizontal shear mode that becomes a torsional one. Thus, the finite dimension of the rod creates three types of resonance: flexural, compressional and torsional. For symmetry reasons, the *SH*_0_ mode in the plate is only coupled to the torsional resonances of the rods, and their coupling falls into the well-studied hybridization effect^20^. From now on, we will focus on the *A*_0_ and *S*_0_ modes that are both coupled to the flexural and compressional resonances of the rods, as will be described.

## Experimental results

**Figure 1:**
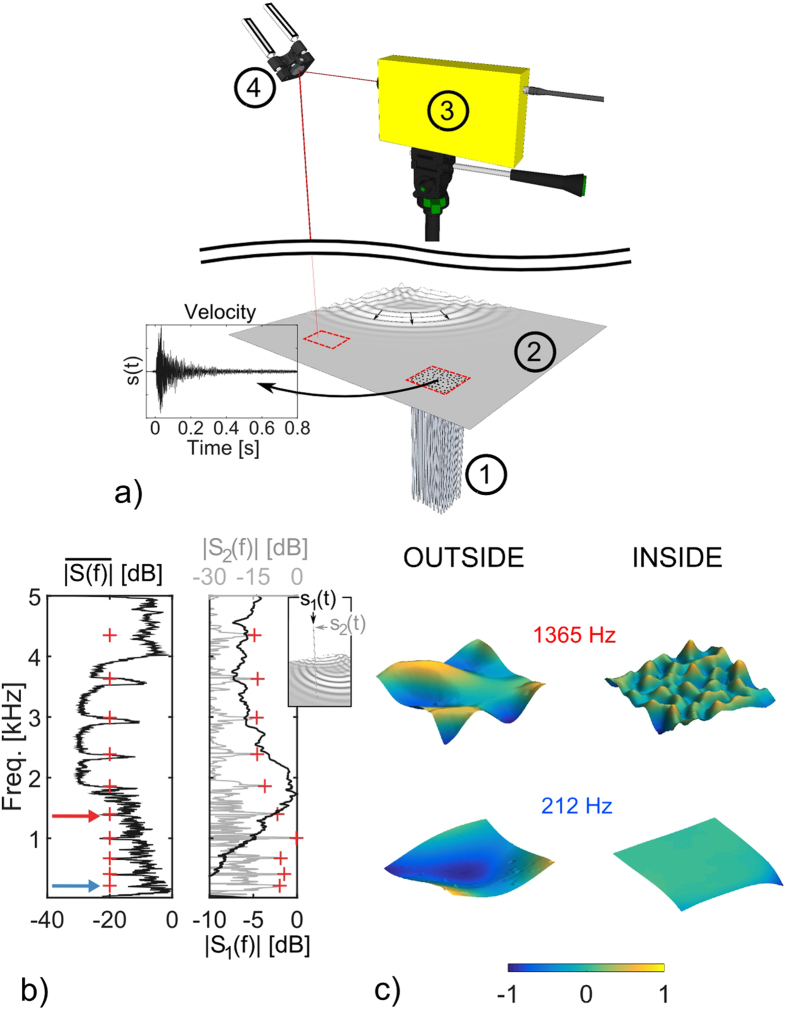
(**a**) The metamaterial consists of a group of 100 aluminum rods (1) that are 61 cm long with a 6.35-mm diameter. These are glued to a thin square aluminum plate (2) of 1.5 m sides, by 2 mm thick. The rods are confined to a square area of 20 cm sides and are randomly arranged. A brief vertical acceleration (impulse) is given to the plate using a shaker as represented by the localized outgoing wavefront at the opposite corner of the plate. The transverse component of the time-domain signal is measured using a laser Doppler velocimeter (3) focused on the plate through a motorized mirror driven by a computer (4). This set-up allows the sequential measurement of impulse responses at any position in the red dashed squares of the 20-cm side indicated on the plate. An example of an impulse response measured at the center of the metamaterial and noted as *s*(*t*) is shown, with strong reverberation on the plate boundaries. (**b**) Left: The average spectrum 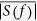 measured at the core of the metamaterial shows a large frequency bandgap between 2 kHz and 4 kHz, in which there are three narrow transmitted bands. The red crosses correspond to the flexural resonances of a single rod. Right: The spectrum measured at the top of one rod for both the compressional resonance (black line, |*S*_1_(*f*)|) and the flexural resonance (gray line, |*S*_2_(*f*)|) when only a single resonator is attached to the plate, as shown in the inset. (**c**) Spatial maps of the wave-field at different frequencies, situated near to a flexural resonance, as indicated by the arrows on the spectrum in (**b**). Red arrow, one frequency where there is a sub-wavelength mode inside the metamaterial; Blue arrow, one frequency situated in a narrow forbidden bandgap with no energy inside the metamaterial.

We start our investigation with the set-up described in Fig. 1a. A broadband *A*_0_ Lamb wave is generated by a vertical shaker placed on the upper side of a thin aluminum plate, while a group of 100 long aluminum rods that form a locally resonant metamaterial is attached on the bottom side of the plate. We perform temporal measurements of the plate motion at different positions inside the metamaterial using a laser Doppler velocimeter (see Methods). The strongly dispersed temporal signals (also called codas; see Fig. 1a, *s*(*t*)) are the consequence of the trapping of the initial pulse in the finite size plate that induces strong reverberation. When performed on the densely sampled grid on the plate, this ensemble of measurements gives spatio-temporal information that contains a great variety of incident wavevectors on the metamaterial.

Looking at the average spectrum measured at the core of the metamaterial in the left panel of Fig. 1b, a wide bandgap between 2 kHz and 4 kHz associated with the compressional resonance of one rod (Fig. 1b, right panel, black line), is observed, a result already explained in an analog experiment^28^. Indeed, there are strong similarities between the present experimental set-up and that studied previously^28^,^29^,^30^ on the effect of a cluster of metallic rods glued at a sub-wavelength scale to a 6-mm thick metallic plate. The main difference lies in the thickness of the plate, which is reduced in the present case to only 2 mm, while the dimensions of the rods and their random spatial distribution remain identical. As a consequence, new phenomena are observed here that were not visible in the previous studies with the more rigid plate. First, three narrow transmitted bands in the frequency interval of the compressional bandgap are clearly associated with the flexural resonances of one rod (Fig. 1b, right panel, gray line), which are indentified by the red crosses in Fig. 1b. Second, before the bandgap at 2 kHz, a few narrow forbidden bands are seen, as well as sub-wavelength modes that are also connected with flexural resonances (Fig. 1c).

As the flexural rigidity of a plate is proportional to the cube of its thickness^31^, this difference has the consequence of rendering the present plate more flexible than the previous 6-mm plate. This is of critical importance for the study of the flexural resonance of the rods. While the effects of the rods were marginal with the 6-mm plate, where the hybridization of the wave-field was mainly due to the compressional resonances of the rods, here it becomes influent enough to be studied in detail.

**Figure 2:**
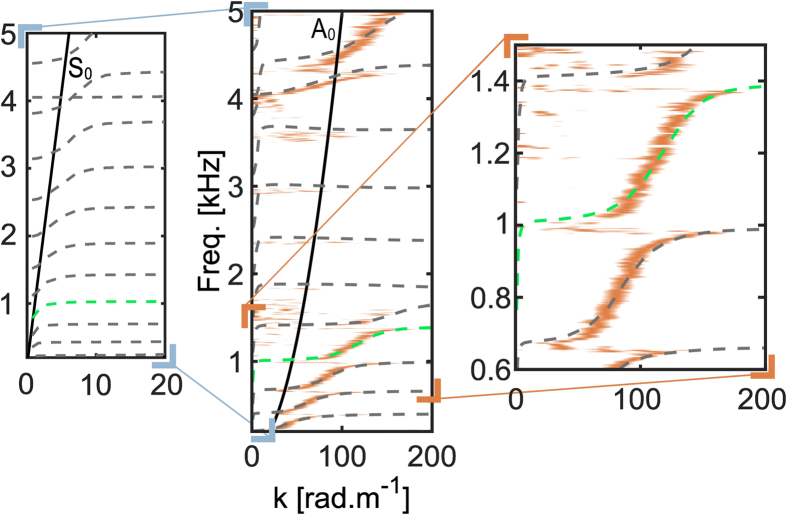
Frequency-wavenumber representation of the dispersion relation inside the metamaterial through a plane wave decomposition. A band structure is revealed that is totally different from the dispersion curve of the flexural waves in the free plate (black curve, *A*_0_). The gray dashed lines are the dispersion curves determined from numerical simulations on the unit cell of an equivalent periodic arrangement of the rods. The propagative bands correspond to the eigenfrequencies of the unit cell with the Bloch periodic boundary conditions determined for each wavenumber of the first Brillouin zone. The left panel is a zoom in on the small wavenumbers that highlight the hybridization effect with the fast *S*_0_ symmetric Lamb waves. Note that only the numerical dispersion curve is represented on these axes for the sake of clarity, as the longitudinal orientation of the field considered is not detected by the experimental set-up. The zoom in for the right panel corresponds to the frequency range of interest for the rest of this report: [600 1500] Hz.

Using the measurements realized between the fixed position of the source and the different receiving locations inside the metamaterial, we experimentally determine the dispersion relation (see Fig. 2 Methods). We observe that the quadratic dispersion relation of the *A*_0_ Lamb waves (Fig. 2, black line) is transformed into a succession of propagative bands, that at every resonance frequency have an asymptotically flat band. This is the polariton behavior that is expected when the free-space dispersion relation crosses a flexural resonance frequency, which was not observed with the more rigid plate. These flat branches correspond to the sub-wavelength modes already discussed in Fig. 1c (top right panel). To obtain more physical insight onto the richness of the measured dispersion relation, we perform further numerical simulations on the unit cell of an equivalent periodic arrangement of rods. Imposing the Bloch periodic boundary conditions at the edges of the unit cell, the eigenvalue problem is solved for a finite number of wavenumbers *k* in the first Brillouin zone (*k* ∈ [0;*π*/*a*], where *a* is the mean inter-rods spacing). The results (Fig. 2, gray dashed lines) are in very good agreement with the measurements, and they validate the use of the simulation for further analysis. Subsequently, as the experimental arrangement is random, this first result means that the band structure is not governed by the periodicity of the scatterers in such a locally resonant medium, as opposed to the case of the phononic crystals where bandgaps originate from the band folding^28^,^32^. Second, although it is hardly noticeable with the experimental dispersion curve, the band structure also catches propagative bands between 2 kHz and 4 kHz. These correspond to the transmission peaks already observed in the experimental data (Fig. 1b). Third, by following a single branch from low to high wavenumbers (e.g., Fig. 2, single green dashed line between the three panels), unusual behavior can be seen: two inflections occur at two consecutive flexural resonances. This strongly differs from typical polariton curves. Instead of giving rise to a hybridization bandgap, the flexural resonances create a propagative band at low *k* (Fig. 2, left panel) which has an inflection along with the longitudinally polarized (in-plane) *S*_0_ dispersion relation. This point is only revealed by the numerical simulations, as the laser probe is only sensitive to the transverse (out-of-plane) component of the wave-field. To obtain further details on the particular effects induced by the flexural resonances, and because the simulation is in very good agreement with experiments, we now follow the study with numerical simulations that allows us notably to access the longitudinal component of the wavefield. Note that for the sake of clarity, we limit the analysis to the bandwidth ranging from 600 Hz to 1500 Hz, which corresponds to the zoom in of the right panel in Fig. 2.

### Numerical results

**Figure 3:**
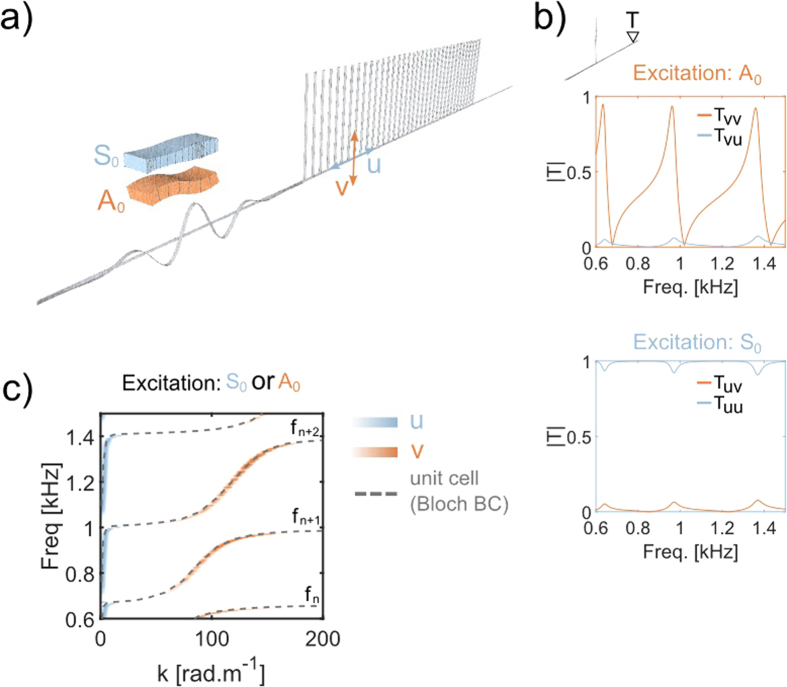
(**a**) Unidimensional system that reproduces the physics of the experimental set-up. The temporal evolution of an initial pulse is calculated using a numerical simulation based on a finite element method. A set of aluminum rods is coupled to a uniaxial plate of 2-mm thickness. Two simulations are performed for two excitation symmetries that correspond to the *A*_0_ and *S*_0_ Lamb modes. The blue color is associated to the longitudinal displacement (*S*_0_), while the transverse displacement that corresponds to the *A*_0_ mode is associated to the orange color. (**b**) Transmission coefficients calculated in the far-field (see position of probe *T* in the upper panel) of a single rod. The top orange axes correspond to an anti-symmetric excitation that produces at point *T* some component on both *u* and *v*, although only the transverse component *v* is initially excited. Similarly, when the excitation is symmetric (bottom blue axes) every flexural resonance creates an energy transfer on the transverse component *v*. (**c**) From the signals extracted from an array inside the rods, we perform a fk transform to retrieve the dispersion curves. The gray dashed lines correspond to the same numerical simulation shown in Fig. 2. This representation aims to compare with the zoom in of the right panel in Fig. 2. It shows the hybrid nature (*u*-polarized for small wavenumbers, and *v*-polarized for large wavenumbers) of the propagative bands caused by the succession of flexural resonances of the rods (the asymptotes of the propagative bands, indicated with *f*_*n*_).

Although the numerical band structure shown in Fig. 2 (dashed gray lines) reveals the atypical hybridization mechanism that occurs in this configuration, it has a severe drawback: the longitudinal and transverse nature of the wave-field cannot be separately identified. To overcome this limitation, we now perform full propagative numerical simulations to recreate the experimental configuration, and thus to observe the entire wave-field. The numerical equivalent (computed with a finite element code) of the experimental set-up is described in Fig. 3a. A broadband *A*_0_ Lamb wave is sent on a one-dimensional periodic arrangement of one or several rods coupled to a uni-dimensional plate (beam). The dimensions and material properties are those used in the experiment (see Methods). The possibility to model the propagation in the bi-dimensional metamaterial with a uni-dimensional system has already been demonstrated by solving the eigenvalue problem on the unit cell, and this is supported by other analytical studies^33^.

Note that from now on, a strict color code is applied: blue is dedicated to the longitudinal component of the wave-field (displacement), indicated with *u*, while orange corresponds to the transverse component of the wave-field, indicated with *v*. In addition, the names of the different transmission coefficients *T* in the following have indices that correspond to the source excitation for the first index (*v* for the *A*_0_ Lamb wave, and *u* for the *S*_0_ Lamb wave) followed by the receiver polarization for the second index. For example, the transmission coefficient indicated as *T*_*uv*_ corresponds to the amplitude of the transmitted transverse component when the initial excitation was longitudinal (*S*_0_). The first interesting result is obtained from the numerical simulation performed for a single rod. The transmission coefficient *T*_*vv*_(*f*) (Fig. 3b) has a succession of characteristic dissymetric Fano interferences^5^ that are induced by the three flexural resonances in this bandwidth. One particularity should be noted: this transmission coefficient is never equal to 1. This is due to the energy transfer that is created by the resonances and revealed by the non-zero values of *T*_*vu*_. This indicates that the flexural resonances create a conversion of the *A*_0_ mode into the *S*_0_ mode, even if they are supposedly orthogonal solutions in thin plates. Similarly, when the incoming wave is *u*-polarized, we observe the same conversion *T*_*uv*_(*f*) from *S*_0_ to *A*_0_. In other words, the modal deformation of the flexural resonances induces a local motion of the plate with components on both *u* and *v*. It is then compatible with both propagative modes of the plate, and stimulates the local coupling between *A*_0_ and *S*_0_. Having established that the flexural resonances convert the wave-field from one component to the other, we now look at the wave-field inside a one-dimensional metamaterial made of 68 rods. The spatio-temporal Fourier transform on both components of the wave-field yields a complete view of the dispersion curve (Fig. 3c). First, Whatever the source excitation (either *A*_0_ or *S*_0_), the propagative modes inside the metamaterial have both polarizations. The small wavenumber part of the propagative modes is polarized along *u*, while the large wavenumber part is polarized along *v*. This is a consequence of the coupling realized by the flexural resonances between the *A*_0_ and *S*_0_ modes in the host medium that, again, strongly differs from the hybridization scheme for single mode scalar waves. Second, there is a transition zone on a narrow frequency band in-between the *u*-polarized and *v*-polarized part of one propagative mode, where the wavefield is a combination of both longitudinal and transverse components. This is at the origin of the transmission peaks we observe experimentally at higher frequencies (between approximately 2 kHz and 4 kHz). Indeed, even in the presence of a wide band gap due to the effect of the compressional resonance of the rods onto the anti-symmetric *A*_0_ mode, the hybrid mode created by each flexural resonance can tunnel through this wide band gap.

**Figure 4:**
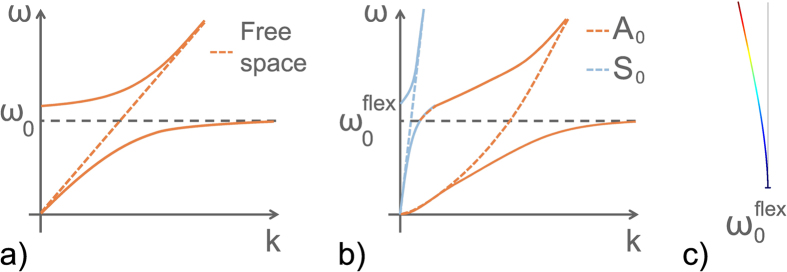
(**a**) Schematic representation of the hybridization effect for a polariton-like configuration: the interaction between a propagating wave (orange dashed line) and a local resonance *ω*_0_ leads to the appearance of two hybrid modes. Below the resonance, the sub-wavelength branch, and above the resonance, the supra-wavelength branch. Both are separated by a bandgap. (**b**) When two orthogonal modes are the solution of the wave equation, and considering a localized resonance where modal deformation induces a local motion of the plate that is compatible with both modes (see (**c**)), the hybridization effect causes the appearance of a third branch. This latter corresponds to a hybrid mode with an evolving symmetry with respect to the wavenumber. The dispersion curves measured experimentally (see Fig. 2) corresponds to the transverse part of this mode, which is repeated at every flexural resonance. (**c**) Representation of the modal deformation of the asymmetric system of plate + resonator at the resonance 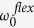 that induces a local longitudinal and transverse deformation on the plate. This modal deformation is responsible for the coupling between the *A*_0_ and *S*_0_ Lamb waves.

To illustrate this particular hybridization effect, Fig. 4 shows two schematic representations for comparisons with the hybridization effects of a scalar wave interacting with a single local resonance (polariton-like behaviour^20^, see Fig. 4a). In this case, the repulsion effect leads to the appearance of two branches, one below and one above the local state. These branches are referred to as the binding and anti-binding branches, respectively, in solid state physics. In wave physics, it is more suitable to talk about sub-wavelength and supra-wavelength branches separated by a bandgap. Although this polariton-like behavior can be slightly modified by strong near-field couplings, it is observed in any locally resonant metamaterials and it has promoted many studies related to negative effective property^4^,^6^,^7^,^8^. However, for a host medium where two vectorial modes can propagate, a metamaterial made of localized resonances that create local deformations with a symmetry compatible with both propagative modes (Fig. 4c), exhibits the dispersion described in Fig. 4b. In this configuration a major change can be noted compared to Fig. 4a as there is a third branch. To fully interpret this dispersion relation, it is important to note that we have broken the natural planar symmetry of the plate by gluing the resonators on a single side. This symmetry violation explains why the flexural resonances locally break the natural orthogonality between *A*_0_ and *S*_0_. As a consequence, a degenerated mode appears that has both longitudinal and transverse polarization. Thus, the succession of propagative bands obtained in Fig. 2b corresponds to the repetition of this type of branch. Note that we deal here with two modes in the plate with two distinct dispersion curves, although this elastic wave property should similarly apply to the case of electromagnetic waves that also show two polarizations, or to multiple modes propagating in waveguides.

**Figure 5:**
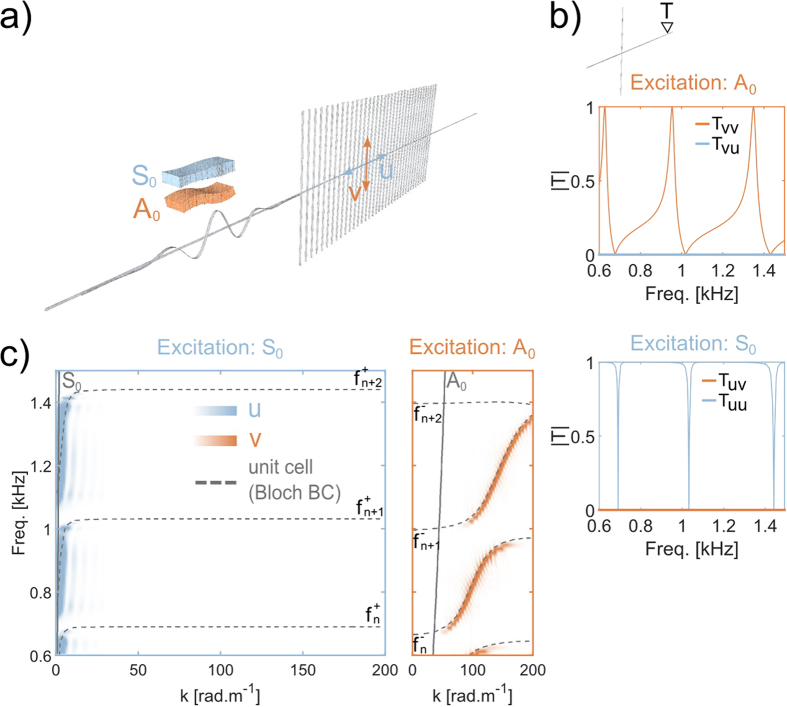
(**a**) Configuration of the numerical simulation with the symmetric metamaterial. The only difference with the asymmetric case (Fig. 3a) is that the rods are distributed on both sides of the plate. (**b**) Transmission coefficients obtained in the far-field of a single unit cell (see sketch at the top). This time, no coupling is observed at point *T*, whatever the initial symmetry of the source. Indeed, the anti-symmetric excitation produces only transverse longitudinal displacement (see top orange axes), while only longitudinal displacement is obtained for symmetric excitation (see bottom blue axes). (**c**) The dispersion curves do not correspond to modes with hybrid symmetry anymore. Both the symmetric (blue) and anti-symmetric (orange) components are independent of each other and require the correct symmetry of excitation (*S*_0_ and *A*_0_, respectively). Contrary to the case of asymmetric metamaterial (see Fig. 3c), every flexural resonance *f*_*n*_ is split into two resonances 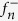 and 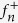 (see Fig. 6, illustration of their modal deformation). Each of these corresponds to a particular symmetry: *A*_0_ for 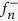, and *S*_0_ for 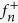.

**Figure 6:**
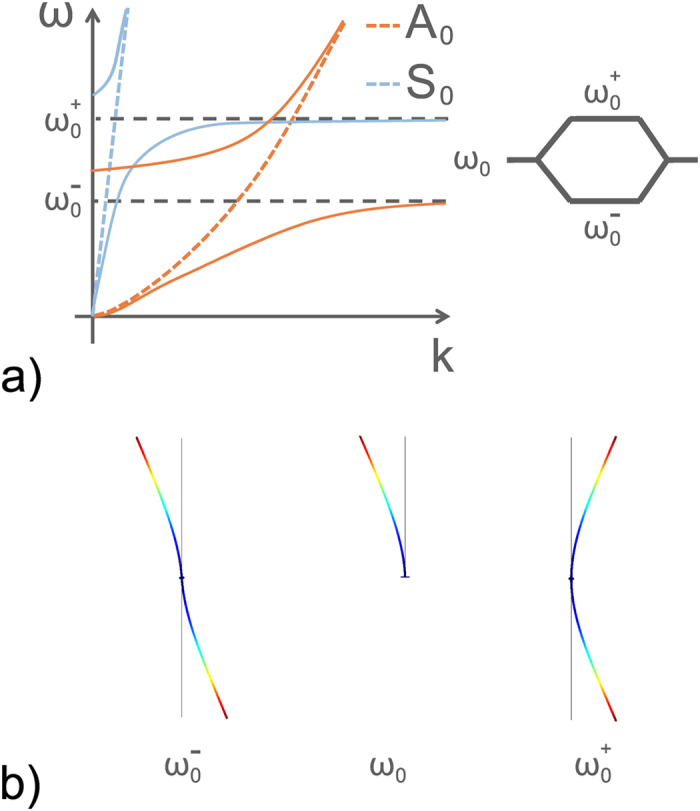
(**a**) Schematic representation of the hybridization observed in the case of a symmetric arrangement of rods on both sides of a thin plate. Two independent polariton-like hybridization curves are observed. As a consequence of this decoupling, the sub-wavelength branch of the longitudinal hybridization overlaps the supra-wavelength branch of the transverse hybridization. Note that these hybridizations occur at two different frequencies 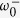 and 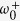 around the fundamental frequency *ω*_0_. (**b**) Representation of the modal deformation of the degenerated modes 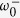 and 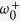. These modes are the consequence of the repulsion effect due to the tight-binding coupling. Then, the flexural deformation *ω*_0_ excited by both the anti-symmetric *A*_0_ and symmetric *S*_0_ modes is replaced by two flexural deformations, each of which corresponds to a particular symmetry of the excitation 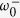 for the *A*_0_ Lamb mode and 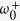 for the *S*_0_ Lamb mode.

As the asymmetry of the system is identified as the origin of the complex band structure measured experimentally, it is now interesting to consider the case where the natural planar symmetry of the plate is recovered. A good way to do this is to place the same arrangement of rods on both sides of the plate (Fig. 5a). Similar to the study of the previous asymmetric system, we look at the transmission coefficients through one rod for two initial excitations that correspond to modes *A*_0_ and *S*_0_ (Fig. 5b). A first significant difference appears: both *T*_*vu*_ and *T*_*uv*_ are null. Whatever the initial symmetry of the excitation, no conversion is observed between *A*_0_ and *S*_0_, which means that the orthogonality is retrieved. As a consequence, both *T*_*vv*_ and *T*_*uu*_ are defined between 0 and 1. To understand the origin of this phenomenon, we now look at the wavefield inside a symmetric metamaterial made of 68 rods. Again, to observe the dispersion of the longitudinal (resp. transverse) component of the wave-field, it is now necessary to use the corresponding symmetric (resp. anti-symmetric) initial excitation. Considering the longitudinal part of the wave-field (Fig. 5c, left panels), only one hybridization is observed around the *S*_0_ dispersion curve, while flat bands are obtained for the large wavenumbers. Similarly, the transverse part of the wave-field (Fig. 5c, right panel) shows a single hybridization around the *A*_0_ dispersion curve. The band structure of the two dispersion relations given in Fig. 5c does not show multi-modal branches anymore: both of the components of the displacement are now independent, and they have to be considered separately. The longitudinal (resp. transverse) component of the displacement associated with the symmetric (resp. anti-symmetric) Lamb mode *S*_0_ (resp. *A*_0_) shows scalar anti-crossing that results from a resonance that must respect the symmetry of the initial excitation. This assertion implies the existence of two uncoupled resonances that are revealed by a relatively subtle detail that is visible on the branches given by the numerical simulations on the unit cell (Fig. 5c, gray dashed lines). Indeed, there is a mismatch between the horizontal asymptotes associated with the respective polarizations. Each *n*^*th*^ resonance is split into two degenerated ones, noted as 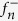 and 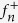. The schematic representation given in Fig. 6a offers better understanding. In this representation, two polariton-like behaviours can be seen. The blue and orange branches can now cross, because of their full decoupling for symmetry reasons. The horizontal asymptotes are not the same and this can easily be explained by a simple model of coupled oscillators. A system of two directly coupled oscillators of eigen-frequency *ω*_0_ has two frequencies 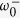 and 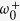. As illustrated in Fig. 6b, the lowest frequency 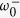 has the symmetry of the *A*_0_ Lamb mode, while the highest frequency 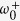 has the symmetry of the *S*_0_ Lamb mode. Thus, the symmetrized resonators give hybridized modes that have the symmetry of the natural modes of the free plate. This configuration allows us to retrieve scalar interactions between a polarized incident wave and local resonances, with the preservation of the orthogonality of the propagative modes.

## Conclusion

In this paper, we define the complex hybridization between the two zero-order elastic plate waves, the *S*_0_ (longitudinal) and *A*_0_ (transverse) Lamb modes, with local flexural resonances. This is realized experimentally and numerically with the study of a system composed of a group of long aluminum rods closely packed and attached to the bottom face of a flexible metallic plate. The difference with a scalar hybridization usually encountered in any locally resonant metamaterials comes from the partial conversion of the transversally (resp. longitudinally) polarized incident wave into a longitudinal (resp. transverse) one. Consequently, the propagative modes resulting from this hybridization have a polarization that is both longitudinal (for the small wavenumbers) and transverse (for the large wavenumbers). This break of orthogonality of the propagative modes in the metamaterial is linked to the asymmetry of the system. Then, when restoring the symmetry by attaching the rods to both sides of the plate, the orthogonality is preserved, which gives rise to two independent scalar hybridization effects for each mode. In this article, we studied the particular case of Lamb waves where the two propagating modes have completely different velocities, although we believe that our approach is valid for any medium that supports two propagating modes at the same frequency. For example, in electromagnetics two modes with different polarizations coexist, and mode conversions need to be thought about when designing a locally resonant metamaterial.

## Methods

### Experimental set-up

The source is a shaker placed on the upper side of the plate, to generate antisymmetric *A*_0_ Lamb waves in the frequency range [150 5000] *Hz*. The measurement is realized by using a laser Doppler velocimeter that points at a motorized mirror, as shown on the top part of Fig. 1a. This set-up allows sequential acquisition of the transverse component of the velocity at any location on the surface of the plate. Due to the vertical orientation of the laser beam, added to the restricted signal-to-noise ratio offered by the laser Doppler velocimeter, the measured wave-field does not include the *S*_0_ Lamb mode, which is mainly longitudinally polarized. We restricted the analysis to the square surface of 20 *cm* side that includes the rods and delimited by the red dashed square in Fig. 1a. The characterization of the wave-field was realized by the measurement of the impulse responses between the fixed source location (at the center of the wave pattern shown on the plate in Fig. 1a) and the different points of the measurement surface, with a spatial resolution of 3 *mm* in both directions. The film of the temporal evolution of the wavefield (spatial maps of the transverse velocity) inside and outside the metamaterial is given in the Supplementary Materials. In addition, we maximized the signal-to-noise ratio in a large frequency band by using a long chirp for the emission, which after cross-correlation, corresponds to an impulse of about 8 *ms*. As revealed by the measured signal indicated as *s*(*t*) and shown in Fig. 1a, the impulse responses obtained inside (or outside) the rods consist of very long reverberated codas of more than 100 times the initial impulse due to the long reverberation time of the plate. Finally, the 100 cylindrical aluminum rods are randomly distributed in a square area of 20 *cm* side and glued vertically on one side of the plate. These have the following dimensions: *L*61 *cm*, 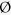 6.35 *mm*.

### Determination of the experimental dispersion relation

The dispersion relation in Fig. 2 is obtained by applying a plane-wave decomposition of the wave-field measured from each point of the square surface inside the metamaterial (Fig. 1a). Thus, all of the monochromatic wave vectors are determined through a two-dimensional fk transform^34^,^35^ and used to estimate the wavenumbers. Due to the strong reverberation on the plate boundaries, the wavefield rapidly becomes isotropic. Thus, the wavenumber determination is made robust by averaging the incoming wave-vectors over 2*π* for every frequency component. The resulting dispersion curve corresponds to the orange branches plotted in Fig. 2.

### Numerical simulations

The configuration described in Figs 3a and 5a are modeled and solved using finite elements software (COMSOL). The rods are similar to the experimental ones, and we used the following properties for aluminum: *ρ* = 2700 *kg* · *m*^−3^, *E* = 69 *GPa*, *ν* = 0.33, where, *ρ*, *E*, *ν* are the volumic mass, the Young’s modulus and the Poisson’s coefficient, respectively. The lattice constant is 1.5 *cm* and the supporting medium is a beam of 2 *mm* thickness made of the same aluminum as the rods. The simulation is solved in the time domain from an initial pulse with a central frequency in the frequency range of interest. Two calculations were performed that corresponded to the two symmetries of the initial excitation: symmetric (*S*_0_ Lamb mode) and anti-symmetric (*A*_0_ Lamb mode).

## Additional Information

**How to cite this article**: Rupin, M. *et al*. Symmetry issues in the hybridization of multi-mode waves with resonators: an example with Lamb waves metamaterial. *Sci. Rep*. **5**, 13714; doi: 10.1038/srep13714 (2015).

## Supplementary Material

Supplementary Information

Supplementary Movie S1
