# Supplementary material for: Symmetry issues in the hybridization of multi-mode waves with resonators: an example with Lamb waves metamaterial
Source: Sci Rep. 2015 Sep 3;5:13714. doi: 10.1038/srep13714 (PMC4558541; doi:10.1038/srep13714)
Supplement: Supplementary Information [file srep13714-s1.doc]

**Symmetry issues in the hybridization of multi-mode waves with resonators: an example with Lamb waves metamaterial**

Matthieu Rupin^1*^, Philippe Roux^2^, Geoffroy Lerosey^1^, and Fabrice Lemoult^1^

^1^Institut Langevin, ESPCI ParisTech and CNRS UMR 7587, PSL Research University, 1 rue Jussieu, 75005, Paris, France

^2^Institut des Sciences de la Terre, UMR 5275, Université Joseph Fourier, Grenoble, 38000, France

^*^matthieu.rupin@espci.fr

**Supplementary Movie**

"Film of the temporal evolution of the out-of-plane component of the velocity. This film compares the wavefield measured at the two areas of the plate schematically represented in Figure 1 (with and without the metamaterial)."
